# Modal Shift from Cars and Promotion of Walking by Providing Pedometers in Yokohama City, Japan

**DOI:** 10.3390/ijerph16122144

**Published:** 2019-06-17

**Authors:** Kimihiro Hino, Ayako Taniguchi, Masamichi Hanazato, Daisuke Takagi

**Affiliations:** 1 Department of Urban Engineering, Graduate School of Engineering, The University of Tokyo, Tokyo 113-8656, Japan; 2 Department of Risk Engineering, Graduate School of Systems and Information Engineering, University of Tsukuba, Ibaraki 305-8573, Japan; taniguchi@risk.tsukuba.ac.jp; 3 Center for Preventive Medical Sciences, Chiba University, Chiba 263-8522, Japan; hanazato@chiba-u.jp; 4 Graduate School of Medicine, School of Public Health, The University of Tokyo, Tokyo 113-0033, Japan; dtakagi-utokyo@umin.ac.jp

**Keywords:** mobility management, public transport, step counts, city planning, compact city, neighborhood

## Abstract

Mobility management is a transportation policy aiming to change travel behavior from car use to sustainable transportation modes while increasing people’s physical activity. Providing pedometers and visualizing step counts, popular interventions in public health practice, may constitute a mobility management program. However, the ease of modal shifts and changeability of walking habits differ across neighborhood environments. Using questionnaire data from 2023 middle-aged and older participants from Yokohama, Japan, in May 2017, this study examined (1) the relationship between the physical and social environments of Yokohama Walking Point Program participants who volunteered to use free pedometers and their modal shifts from cars to walking and public transport, and (2) whether participants’ modal shifts were associated with increases in step counts. Multivariate categorical regression analyses identified the frequency of greetings and conversations with neighbors as well as health motivation as important explanatory variables in both analyses. Participants living in neighborhoods far from railway stations and in neighborhoods with a high bus stop density tended to shift to walking and public transport, a modal shift that was highly associated with increased step counts. These results suggest that mobility management should be promoted in collaboration with public health and city planning professionals.

## 1. Introduction

Although considerable evidence exists demonstrating that physical inactivity increases the risk of major non-communicable diseases and shortens life expectancy, much of the world’s population is inactive [1,2]. Recently, researchers have urged city planning policies to increase opportunities for physical activity (PA) by encouraging active transport (e.g., walking and cycling) and public transport use (e.g., railway and local bus) and reducing private car use [3,4,5]. One such policy concerns the creation of compact cities in which major facilities are concentrated within the city center, around public transport hubs, enabling residents to walk to public transport. This model is in contrast to sprawled cities in which residents are dependent on cars [5,6].

The Japanese government at the national and municipal levels have sought to promote compact city policies in light of the need to reduce environmental load and the reality of an increasing older population. The Japanese city of Toyama’s city planning policies are reflective of the trend towards compact cities, with the municipality promoting public transit-oriented development and vitalization through initiatives such as the opening of the first light-rail transit in Japan and the subsidizing of costs for the acquisition of dwellings in the city center and along public transport lines [7,8]. Nonetheless, the realization of a truly compact city requires tens of years to come to fruition; as such, it is necessary to implement policies beyond those that look to change the physical environment, policies that promote a modal shift from cars to walking and public transport (hereafter simply referred to as “modal shift”), in addition to policies that look to reshape the urban structure.

Mobility management (MM) is one example of such non-physical transportation management policies. MM aims to change travel behaviors from car use to sustainable transportation modes (i.e., public transport and active transport) using communicative measures such as the provision of specific information on public transport, travel education, and word-to-mouth recommendation [9,10]. We use the term MM according to these definitions hereafter in this study, while MM is often referred to as travel planning in the United Kingdom [11] and voluntary travel behavior change in Australia [12]. Drawing on research from social and environmental psychology, studies on MM have accumulated since the mid-1990s, with such research contributing to the identification and development of effective methods of promoting modal shifts [13]. In addition, factors that influence the choice of travel mode, such as travel time and family structure, have been investigated [14,15,16].

MM practices have been reported not only in developed but in developing countries as well. In Metropolitan Manila, Philippines, a rideshare app for university students was developed to promote behavioral change [17,18]. In Japan, typical MM practices include personal conversations, workshops, education initiatives in schools, and travel feedback programs, practices that look to address social problems caused by car overuse [19]. A MM program in Yamato, Kanagawa, in which participants were provided pedometers in addition to leaflets and town guides, succeeded in decreasing their car use and increasing their PA [20].

In public health research and practice, providing pedometers and visualizing step counts is also a popular intervention for promoting PA [21,22,23]. Compared to other devices used to visualize step counts, a pedometer is cheaper and easier for every population to use. A systematic review of studies which assessed pedometer use among adults suggests that pedometer use is associated with significant increases in PA and improvements in several key health outcomes [24]. However, the ease with which modal shifts and the changeability of walking habits occur differs according to participants’ neighborhood environments. Several studies have shown that public transport users spend greater amounts of time walking [25,26], with access to public transport associated with increased PA [27,28,29] and walking [30,31]. For example, an analysis of 6822 adults from 14 cities in 10 countries found that public transport density is significantly, positively, and linearly correlated with increased PA [32]. In addition, a longitudinal study confirmed that access to bus stops and railway stations is a key determinant of walking as a mode of transportation [33]. Based on existing studies demonstrating a relationship between public transport and PA/walking habits, the ease with which modal shifts and changeability of walking habits occur must be analyzed in light of participants’ access to public transport in order for effective intervention to occur.

This study engages in such an analysis within the context of a program in the Japanese city of Yokohama, in which participants volunteered to use free pedometers to promote PA and improve their health. The first part of this study examines the relationship between participant attributes and their surrounding physical environments (i.e., distance to the nearest railway stations and bus stop density) and social environments (i.e., frequency of interaction with neighbors) on one hand, and modal shift on the other. In the second part of this study, we explore if and how modal shifts are associated with increases in step counts while controlling for other factors. This study contributes to the existing literature by identifying how neighborhood environments influence middle-aged and older people’s active behaviors in the context of a super-aged society such as Japan, which has the world’s highest proportion of older adults among its population [34].

## 2. Materials and Methods

### 2.1. Yokohama Walking Point Program

Located 30–40 km from Tokyo, Yokohama is the second-most populous city in Japan and was developed as an international port city. The city has a population of approximately 3.7 million people, of whom 24% are 65 years or older as of January 2017. The city’s railway network has been developed, with many lines running towards central Tokyo. The railway is approximately 308 km long, and there are 157 railway stations in Yokohama. The local bus network has been expanded around the railway stations, enabling approximately 90% of citizens access to the railway stations within 15 minutes. According to the latest Person Trip Survey from 2008, railway and local bus use constituted 33.9% and 5.8% of the main modes of transportation, respectively, with these figures being higher than in other nearby major cities [35]. Nevertheless, approximately 20% of greenhouse gas emissions in Yokohama are caused by the transportation sector, half of which can be attributed to private cars [36]. Thus, shifting from private cars to public transport is one of the policy targets of the city as it looks to reduce its environmental footprint, improve the sustainability of public transport, and promote citizen health [36].

In November 2014, the city launched the Yokohama Walking Point Program (YWPP) to encourage citizens to improve their health and healthy life expectancy, as the average age of the population and the nature of diseases change. It provided free pedometers (Omron HJ-326F, Japan), purchased with the city budget, for volunteer participants aged 40 years and above. In June 2016, participation qualification was expanded to citizens aged 18 years and above. Participants were awarded points based on their step counts by scanning their pedometers via special readers installed at approximately 1000 stores and other facilities in the city. Accumulation of a certain number of points made participants eligible to win prizes. The scanned data were sent to a data server through the Internet, and participants could monitor step counts and rank among all participants using a computer or smartphone [37,38]. Every time the average monthly step counts from all participants exceed a set target, 200,000 yen is donated to the United Nations World Food Programme.

### 2.2. Data Collection

Participants’ sex, age, neighborhood-level address, and number of months participating in YWPP were acquired from the YWPP registration information. Distance to the nearest railway station was measured from the center of each neighborhood, and bus stop density was calculated for each neighborhood using data from the National Land Numerical Information download service [39].

The other data were measured in the questionnaire survey that Yokohama city conducted in May 2017 among 6000 participants selected from 231,600 participants. They were randomly and proportionally selected from three stratified groups by data sending rate: participants whose data sending rate was 80% or more, less than 80%, and those who never sent data. Among the selected participants, 3493 replied to the survey, with a response rate of 58.2%. Since the original age eligibility requirement for the program was 40 years and older until June 2016, 141 respondents were aged below 40 and were thus excluded from analysis. Ultimately, a total of 2023 participants who answered all necessary questions were included in the survey analysis. The questionnaire asked primarily about the participants’ changes in walking habits and health attitudes as well as their modal shifts after participating in the program for a period of time. Translated questions asked in the survey are presented in Table A1.

### 2.3. Variables

#### 2.3.1. Outcome Variables

In the first analysis, modal shift with four options, ranging from “Yes” to “No”, served as the outcome variable. In the second analysis, increases in step counts served as the outcome variable. Although it originally had four options, ranging from “increased” to “decreased”, “decreased” was selected by only 0.5% of participants and thus merged with “not changed”.

#### 2.3.2. Explanatory Variables

The explanatory variables in both analyses were distance to the nearest railway station, bus stop density, and frequency of greetings and conversation with neighbors. Participants’ modal shift, which was the outcome variable in the first analysis, was added as an explanatory variable in the second analysis.

As the number of bus stops (2735) was much higher than the number of neighborhoods (758) in Yokohama, we used bus stop density rather than distance to a bus stop as an explanatory variable. The mean distance to the nearest railway station was 807.6 (±634.6) m and the mean bus stop density per km^2^ was 7.6 (±9.3). The two variables were disaggregated into four categories by three thresholds: approximately the mean and the mean ± 1/2 SD.

Frequency of greetings and conversation with neighbors was surveyed with five options ranging from “increased” to “decreased”. Not only physical but social features of neighborhoods can also affect health by constraining or enhancing health-related behaviors [40]. Also, relationships between neighbors have been shown to have a positive association with engagement in PA [41].

#### 2.3.3. Control Variables

The control variables in both analyses were participants’ sex, age, occupation, self-rated health, diagnosis of a metabolic syndrome prior to participation, motivation for participation in YWPP, and months participating in YWPP.

Participants’ ages were categorized as non-older adults (<65 years), early-stage older adults (65–74 years), and later-stage older adults (>75 years), based on categories provided by the long-term care insurance system in Japan [42]. Self-rated health [43,44,45], pre-existing metabolic syndromes [46,47], and motivation [48,49] were found to be associated with walking/PA in previous studies. Months participating in YWPP was considered because the effect of pedometers on participants’ walking levels might vary over time [50].

Regarding motivation for participation, we identified four categories—health motivation, profit motivation, data confirmation motivation, and interaction motivation—based on the result of a hierarchical cluster analysis of the original 10 hypothesized options from which participants could select multiple answers (Table A2).

Survey options selected by a small percentage of participants were merged into four variables—occupation, self-rated health, frequency of greetings and conversations with neighbors, and change in step counts—as shown in Table 1.

### 2.4. Statistical Analysis

Multivariate categorical regression was used in both analyses. Categorical regression quantifies categorical variables using optimal scaling and assigns numerical values to categories. It simultaneously scales nominal, ordinal, and numerical variables and treats quantified categorical variables in the same way as numerical variables. Scaling all variables at the numerical level results in standard multiple regression analysis of the transformed variables [51]. In our analyses, all variables including outcome variables were transformed into numerical variables.

The output of the analysis comprises regression coefficients, their statistical significance, and Pratt’s relative importance measure of predictors, which is large for predictors that are crucial to the regression and useful in interpreting predictor contributions to the regression, for all explanatory and control variables [51].

The significance level was set at *p* < 0.05. All statistical analyses were conducted using IBM SPSS Statistics 23 (IBM Corp., Armonk, NY, USA).

## 3. Results

### 3.1. Sample Statistics

Participant characteristics and questionnaire results are presented in Table 1. Regarding outcome variables, more than half of the participants disclosed that they had “shifted from cars to public transport/slightly shifted”, and approximately two-thirds of participants reported that their step counts had “increased/slightly increased” after participation in YWPP. Approximately 40% reported that their frequency of greetings and conversations with neighbors “increased/slightly increased”.

Regarding the control variables, males constituted 43.0% of the sample. The mean age of the participants was 65.7 (±11.1) years, with 61.4% being more than 65 years old as of the end of May 2017. Only 18.1% of the participants had full-time jobs, reflecting the old age of the sample population. Approximately 90% reported that prior to participation in the program, they had been “healthy/rather healthy”, with less than 25% having been diagnosed with a metabolic syndrome. More than half had participated in YWPP for more than 24 months at the time of the survey. As the motivation for participation in YWPP, health, profit, data confirmation, and interaction were selected by 79.4%, 63.4%, 46.7%, and 28.4% of the participants, respectively.

### 3.2. Modal Shift

The left side of Table 2 shows the results of the first analysis, and Table 3 shows the numerical values assigned to the categorical variables. The variables with the most and the second-most importance were, respectively, health motivation and frequency of greetings and conversations with neighbors.

**Figure 1:**
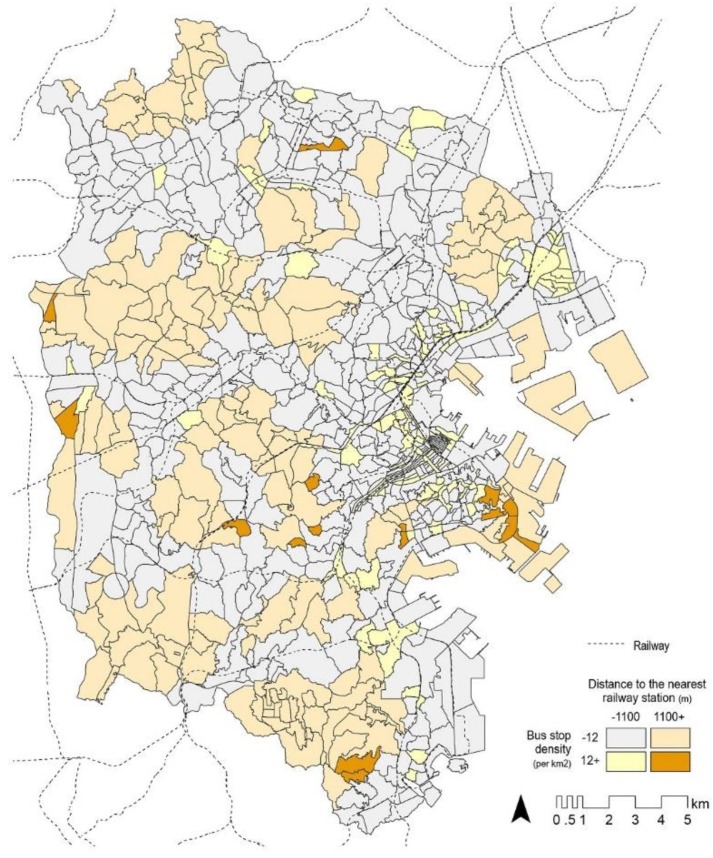
Spatial distribution of accessibility to public transport.

Regarding physical environment, distance to the nearest railway station as well as bus stop density were also significantly associated with the outcome variable. Participants living in neighborhoods far from railway stations (more than 1100 m) and those with a high bus stop density (more than 12 per km^2^) tended to shift from cars to public transport. Figure 1 shows the spatial distribution of such neighborhoods—neighborhoods far from railway stations were located in suburban hilly areas and coastal industrial zones, while most neighborhoods with a high bus stop density were located near city centers.

Regarding the other control variables, being male, unemployed, diagnosed with a metabolic syndrome before participation, and longer months participating in YWPP were positively associated with modal shifts, while age and self-rated health were not. The adjusted R^2^ of the regression was 0.129.

### 3.3. Change in Step Counts

The right side of Table 2 shows the results of the second analysis, with Table 3 showing the numerical values assigned to categorical variables. The variable with the most importance by far was modal shift. This was followed by health motivation and by frequency of greetings and conversations with neighbors, both of which were highly associated with the outcome variable in the first analysis.

Regarding physical environment, bus stop density was significantly associated with increased step counts. Those living in neighborhoods more than 1100 m away from the nearest railway station tended to increase their step counts, although the variable was not statistically significant.

Regarding the other control variables, ages between 65 and 74 years, non-workers, and interaction motivation were positively associated with increased step counts, while sex, self-rated health, and months participating in YWPP were not. The adjusted R^2^ of the regression was 0.260, which was higher than the first analysis.

## 4. Discussion

This study examined aspects of the physical and social environments of middle-aged and older participants, who volunteered to use free pedometers, that are associated with modal shifts and increases in step counts in Yokohama, Japan. Although the adjusted R^2^ of the regression was not high in the two analyses, eight and seven variables were statistically significant in the respective analyses.

The results of the first analysis showed that participants living in neighborhoods far from railway stations and in neighborhoods with a high bus stop density tended to engage in modal shifts. This shift may have occurred because local buses are used for shorter trips than railways, and participants changed their short trip transport mode from cars to buses. On the other hand, participants living near railway stations seemed unable to change their transport mode while participating in the program. This may be because these participants had used cars less frequently than suburban participants prior to participation because of the general features of the city’s transit-oriented development, such as “less convenience for cars and special consideration for pedestrians” [26].

The second half of the study showed that participants’ modal shifts were most associated with increases in their step counts. These results suggest that participation in YWPP promoted modal shifts and walking instead of driving. As seen with modal shifts, living in neighborhoods with a high bus stop density tended to increase participants’ step counts. Although not statistically significant, participants living in neighborhoods far from railway stations tended to increase their step counts. Particularly in light of the results of a previous study on a Japanese rural city that demonstrated that distance to bus stops had significant relationship with PA, but distance to railway stations did not [52], future studies should explore the role of public transport not only by distinguishing railway and bus modalities, but urban and rural environmental differences as well.

Participants’ frequency of greetings and conversations with others, as a proxy for social environment, was positively associated with modal shifts and increases in step counts. Considering the causal relationship, identified by a quasi-longitudinal study [53], in which changes in socializing with neighbors had a positive impact on walking activities, the provision of pedometers in our study might encourage opportunities for communication with other participants and promote participants to go out and use public transport together.

With regard to control variables, motivation to participate in YWPP must be highlighted. Health motivation had a positive association with both modal shift and increased step counts, while self-rated health before participation was not statistically significant. Interaction motivation was also positively associated with increased step counts, a finding potentially driven by the fact that expanded social connections may have contributed to increased frequencies of going out, as discussed above. On the other hand, the other categories of motivations―profit motivation and data confirmation motivation―were not found to be statistically significant in either analysis. These aspects of YWPP appear to not be useful in promoting modal shifts and increased walking.

While participants with preliminary metabolic syndromes tended to report relatively high levels of modal shifts, those with metabolic syndrome did not do so. This result is consistent with previous studies demonstrating that the conditions of overweight and obesity are causally associated with future inactivity [54,55]. These findings suggest the importance of intervention at an early stage because PA promotion for obese and overweight people is more difficult. Considering the fact that health motivation was found to be positively associated with both modal shifts and increased step counts, providing pedometers and PA education during health examinations may be effective at preventing and treating metabolic syndrome.

In Japan, many municipalities list health promotion as a benefit of compact cities as well as the reduction of environmental load [6]. In depopulated areas, however, bus routes may decrease in frequency or even withdraw from these areas entirely, a reality that is undesirable from the viewpoint of health promotion. As such, bus routes and availability should be sustained. During the realization of compact cities, non-physical policies such as MM programs should be promoted in the short-term, informed by collaboration between public health and city planning professionals. Pedometer intervention may prove to be a critical component of such policies, one that contributes to a healthy active urban population.

While making an important contribution to existing research on neighborhood environments and people’s active behaviors, this study has some limitations. Retrospective questions on participants’ public transport use, step counts, self-reported health, and communication frequency with neighbors may be subject to recall bias. Changes in step counts reported in our questionnaire might also be subject to self-reporting bias, as we lacked measures of step counts before the pedometer intervention. In addition, study participants may not be representative of the general population of Yokohama city in that they were motivated to have pedometers, suggesting a higher baseline interest in PA than others. We cannot know precisely the effect of pedometers in reality as in a control experiment due to the absence of comparison with non-participants. Lastly, only the physical environment of neighborhoods in which participants lived was considered in this study. Future studies should examine more detailed factors for modal shifts and the promotion of walking through pedometer use by considering physical and social environments not only around participant homes, but workplaces and favorite places as well. This may be done using travel diary data or GPS data.

## 5. Conclusions

This study examined the relationship between the physical and social environments of middle-aged and older YWPP participants who volunteered to use free pedometers, and their modal shifts from cars to walking and public transport. This study further considered if participants’ modal shifts were associated with increases in their step counts using questionnaire data. The results of multivariate categorical regression analysis find that the frequency of greetings and conversations with neighbors as well as health motivation are highly associated with modal shift. Regarding physical environment characteristics, participants living in neighborhoods far from railway stations and in neighborhoods with a high bus stop density tended to shift to walking and public transport. In addition, modal shift was by far the most associated with increased step counts. This study’s results suggest that pedometer intervention could be an effective component of MM programs that promote healthier, active cities.

## Figures and Tables

**Table 1:** Characteristics of the study samples and questionnaire results (*n* = 2023).

Variables	Options	*n*	%
**Control variables**			
Sex	Male	869	43.0
	Female	1154	57.0
Age (years)	<65	781	38.6
	65–74	778	38.5
	75+	464	22.9
Occupation	Full-time	367	18.1
	Part-time/self-employed	407	20.1
	Non-worker/other	1249	61.7
Self-rated health (before participation)	Healthy	646	31.9
	Rather healthy	1158	57.2
	(Rather) unhealthy	219	10.8
Diagnosis of metabolic syndrome (before participation)	Yes	262	13.0
	Preliminary	238	11.8
	No	1523	75.3
Motivation for participation in YWPP (Multiple Answers)	Health	1607	79.4
	Profit	1282	63.4
	Data confirmation	945	46.7
	Interaction	574	28.4
Months participating in YWPP	<12	421	20.8
	12–24	512	25.3
	24+	1090	53.9
**Explanatory variables**			
Frequency of greetings and conversations with neighbors	Increased	195	9.6
	Slightly increased	621	30.7
	Not changed/(slightly) decreased	1207	59.7
Distance to the nearest railway station	<500 m	583	28.8
	500–800 m	556	27.5
	800–1100 m	286	14.1
	1100 m+	598	29.6
Bus stop density (per km^2^)	<3	330	16.3
	3–7.5	969	47.9
	7.5–12	534	26.4
	12+	190	9.4
**Outcome variables**			
Modal shift	Yes	548	27.1
	Mostly yes	578	28.6
	Mostly no	432	21.4
	No	465	23.0
Change in step counts	Increased	910	45.0
	Slightly increased	408	20.2
	Not changed/decreased	705	34.9

**Table 2:** Results of categorical regression.

Outcome Variables		Modal Shift				Change in Step Counts			
		B	*p*		Importance	B	*p*		Importance
Sex		0.048	0.031	*	0.013	0.026	0.147		0.002
Age (years)		0.040	0.082		0.046	0.067	0.000	***	0.020
Occupation		0.056	0.007	**	0.057	0.062	0.001	**	0.033
Self-rated health ^a^		−0.001	1.000		0.000	−0.039	0.141		0.007
Diagnosis of metabolic syndrome ^a^		0.043	0.009	**	0.019	0.016	0.294		0.002
Motivation:	Health	0.203	0.000	***	0.383	0.158	0.000	***	0.172
	rofit	0.029	0.136		0.011	0.017	0.260		−0.001
	Data confirmation	0.036	0.079		0.007	0.027	0.123		0.004
	Interaction	0.030	0.113		0.020	0.059	0.003	**	0.030
Months participating in YWPP		0.057	0.001	***	0.036	0.018	0.223		0.005
Frequency of greetings and conversations with neighbors		0.199	0.000	***	0.375	0.103	0.000	***	0.097
Distance to the nearest railway station		0.038	0.008	**	0.014	0.019	0.139		0.002
Bus stop density (per km^2^)		0.051	0.000	***	0.019	0.035	0.006	**	0.003
Modal shift						0.369	0.000	***	0.623
*p*		0				0			
Adjusted R^2^		0.129				0.260			

B: regression coefficient (beta); *p*: statistical significance of coefficient (* < 0.05, ** < 0.01, *** < 0.001); importance: Pratt’s relative importance measure of predictors; ^a^ before participation.

**Table 3:** Numerical values assigned to the categories.

Variables	Categories	Outcome Variables	
		Modal Shift	Change in Step Counts
Sex	Male	−1.152	−1.152
	Female	0.868	0.868
Age (years)	<65	1.261	0.352
	65–74	−0.810	−1.168
	75+	−0.764	1.366
Occupation	Full-time	1.684	1.310
	Part-time/self-employed	0.807	1.234
	Non-worker/other	−0.758	−0.787
Self-rated health ^a^ (before participation)	Healthy	−0.598	−0.390
	Rather healthy	−0.200	−0.325
	(Rather) unhealthy	2.823	2.869
Diagnosis of metabolic syndrome (before participation)	Yes	−1.157	1.468
	Preliminary	−2.254	−2.453
	No	0.551	0.131
Motivation			
Health	Yes	−0.509	−0.509
	No	1.965	1.965
Profit	Yes	0.760	−0.760
	No	−1.315	1.315
Data confirmation	Yes	−1.068	−1.068
	No	0.936	0.936
Interaction	Yes	−1.589	−1.589
	No	0.629	0.629
Months participating in YWPP	<12	1.824	0.975
	12–24	−1.061	1.164
	24+	−0.206	−0.923
Frequency of greetings and conversations with neighbors ^a^	Increased	−2.455	−2.027
	Slightly increased	−0.614	−0.883
	Not changed/(slightly) decreased	0.713	0.782
Distance to the nearest train station	<500 m	1.054	0.460
	500–800 m	0.516	−0.380
	800–1100 m	−0.167	2.008
	1100 m+	−1.428	−1.056
Bus stop density (per km^2^)	<3	1.613	-0.986
	3–7.5	−0.156	0.506
	7.5–12	0.154	0.607
	12+	−2.436	−2.573
Modal shift ^a^	Yes	−1.394	−1.146
	Rather yes	−0.191	−0.498
	Rather no	0.655	0.503
	No	1.272	1.503
Change in step counts ^a^	Increased		−0.887
	Slightly increased		−0.334
	Not changed/decreased		1.338

^a^ The order of the categories is preserved in the optimally scaled variables.

## Appendix A

**Table A1:** Questions asked in the survey.

Variables	Questions Asked in the Survey
Self-rated health (before participation)	How did you feel about your health? (before participation)
Diagnosis of metabolic syndrome (before participation)	Have you been diagnosed with metabolic syndrome in a periodic health examination or a medical examination? (before participation)
Frequency of greetings and conversations with neighbors	Did the frequency of greetings and holding conversations with neighbors increase compared to the frequency pre-participation in YWPP?
Modal shift	Did the frequency of walking or using public transport increase while decreasing the frequency of car or motor bike use when going out (e.g., commuting and going shopping) after participating in YWPP?
Change in step counts	Did your daily step count change after participating in YWPP?

**Table A2:** Motivation for participation in YWPP (multiple answer).

Categories	Options	*n*	%
Health motivation	Can promote health while enjoying	1406	69.5
	Good chance to begin walking	666	32.9
	Can feel healthy	261	12.9
Profit motivation	Can get a pedometer	926	45.8
	Can win prizes	715	35.3
	Can donate	255	12.6
Data confirmation motivation	Can confirm data of step counts and rank	945	46.7
Interaction motivation	Can walk with families and friends	502	24.8
	Can interact with other participants	125	6.2
(None)	Can participate with office colleagues	41	2.0

Measure: Phi 4-point correlation, cluster method: centroid clustering. Nine options were categorized into four using hierarchical cluster analysis. The last option (Can participate with office colleagues) was not included in any category because it was not clustered with any of the other nine options and its selection rate was the lowest. When a participant selected any options in category A, the binary variable of the category A was 1.
